# Forest production predicted from satellite image analysis for the Southeast Asia region

**DOI:** 10.1186/1750-0680-8-9

**Published:** 2013-09-10

**Authors:** Christopher Potter, Steven Klooster, Vanessa Genovese, Cyrus Hiatt

**Affiliations:** 1 NASA Ames Research Center, Moffett Field, CA, USA; 2 California State University Monterey Bay, Seaside, CA, USA

**Keywords:** Southeast Asia, Forest production, Harvested wood products, Carbon cycle, MODIS

## Abstract

**Background:**

The objective of this study was to demonstrate a new, cost-effective method to define the sustainable amounts of harvested wood products in Southeast Asian countries case studies, while avoiding degradation (net loss) of total wood carbon stocks. Satellite remote sensing from the MODIS sensor was used in the CASA (Carnegie Ames Stanford Approach) carbon cycle model to map forest production for the Southeast Asia region from 2000 to 2010. These CASA model results have been designed to be spatially detailed enough to support carbon cycle assessments in different wooded land cover classes, e.g., open woodlands, wetlands, and forest areas.

**Results:**

The country with the highest average forest net primary production (NPP greater than 950 g C m^-2^ yr^-1^) over the period was the Philippines, followed by Malaysia and Indonesia. Myanmar and Vietnam had the lowest average forest NPP among the region’s countries at less than 815 g C m^-2^ yr^-1^. Case studies from throughout the Southeast Asia region for the maximum harvested wood products amount that could be sustainably extracted per year were generated using the CASA model NPP predictions.

**Conclusions:**

The method of using CASA model’s estimated annual change in forest carbon on a yearly basis can conservatively define the upper limit for the amount of harvested wood products that can be removed and still avoid degradation (net loss) of the total wood carbon stock over that same time period.

## Background

Net primary production (NPP) by vegetation provides the chemical energy that drives most biotic processes on Earth. NPP represents much of the carbon that is consumed by microbes and animals. Climate controls on plant production are an issue of central relevance to society, mainly because of concerns about the extent to which NPP in managed ecosystems can provide human populations with adequate forage and fiber products. In areas where NPP is not harvested for human use, the carbon in dead plant biomass naturally returns to the atmosphere mainly as CO_2_ from decomposition by microbial respiration.

As a monitoring tool for forest management projects, annual NPP flux of atmospheric carbon (as CO_2_) into standing wood pools represents the yearly increment added to a managed area, which may also include wood harvest activity and extraction of older forest carbon. Allocation of carbon to standing wood pools in tropical forests measured worldwide was found to average 39% ± 10% of NPP flux [1]. When totaled over a defined project boundary area, an increasing trend in annual NPP and wood C turnover time favors net storage of atmospheric CO_2_ within a forest ecosystem (Luo et al. [2]).

In this study, we summarized results from the CASA (Carnegie-Ames-Stanford Approach) model for forest ecosystem NPP across all the countries of Southeast Asia region from 2000 to 2010. The main purpose of the study was to define the upper limit for the amount of harvested wood products that can be removed by logging in selected case studies and still avoid degradation (net loss) of the total wood carbon stock. Direct input of satellite vegetation index “greenness” data from the NASA moderate resolution imaging spectroradiometer (MODIS) satellite sensor into the CASA simulation model was used to estimate spatial variability in annual NPP at a ground resolution of 8-km [3,4]. These CASA model results have been designed to be spatially detailed enough to support carbon cycle assessments in different wooded land cover classes, e.g., open woodlands, wetlands, and forest areas.

### Regional study area

Both Potter et al. [5] and Zhao and Running [6] estimated that the Southeast Asia region accounted for over 25% of all NPP in the tropical rainforest zones globally, with a total regional NPP flux of around 3.7 Pg (10^15^ g) C per year. Miettinen et al. [7] reported an overall 1.0% yearly decline in forest cover in insular Southeast Asia between 2000 and 2010, with the main change trajectories to plantations and secondary vegetation. Peat swamp forests experienced the highest deforestation rates at an average annual rate of 2.2%, while lowland evergreen forest area declined by 1.2% per year.

Among the 30 leading countries listed for area of forest loss annually by the Food and Agriculture Organization (FAO) of the United Nations and by Potter et al. [8], seven of these nations were located in the Southeast Asia region, namely Myanmar, Indonesia, Malaysia, Vietnam, Cambodia, Laos, and Papua New Guinea (in order of highest to lowest). These countries, along with the Philippines and Thailand, comprise the Southeast Asia region for the present study.

## Results and discussion

All of the countries in the Southeast Asia region had 75% or greater of their total forest and open woodland areas classified in the CASA model as evergreen broadleaf forest types, with the exception of Cambodia (at 61%). Indonesia, Laos, Malaysia, Papua New Guinea, and the Philippines each had over 92% of their total forested land classified as evergreen broadleaf types, which put a focus on comparison of NPP in this forest class among the countries listed in Table 1.

**Table 1:** CASA model predictions of mean annual NPP from 2000 to 2010 for Southeast Asia countries by forest, savanna, and wetland land cover classes

	**Evergreen broadleaf forests**	**Evergreen needleleaf forests**	**Mixed forests**	**Open woodlands and savannas**
**Country**				
Myanmar	782	614	663	638
Cambodia	861	726	383	742
Indonesia	953	547	612	747
Laos	878	837	737	761
Malaysia	970	443	882	903
Papua New Guinea	916	818		757
Philippines	1108	662	968	989
Vietnam	812	490	714	727
Thailand	862	704	647	718

All fluxes were reported in units of g C m^-2^ yr^-1^.

Results of the CASA model indicated that the country with the highest average forest NPP (greater than 950 g C m^-2^ yr^-1^) over the period 2000 to 2010 was the Philippines, followed by Malaysia and Indonesia (Table 1). Myanmar and Vietnam had the lowest average forest NPP among the region’s countries (less than 815 g C m^-2^ yr^-1^).

**Figure 1:**
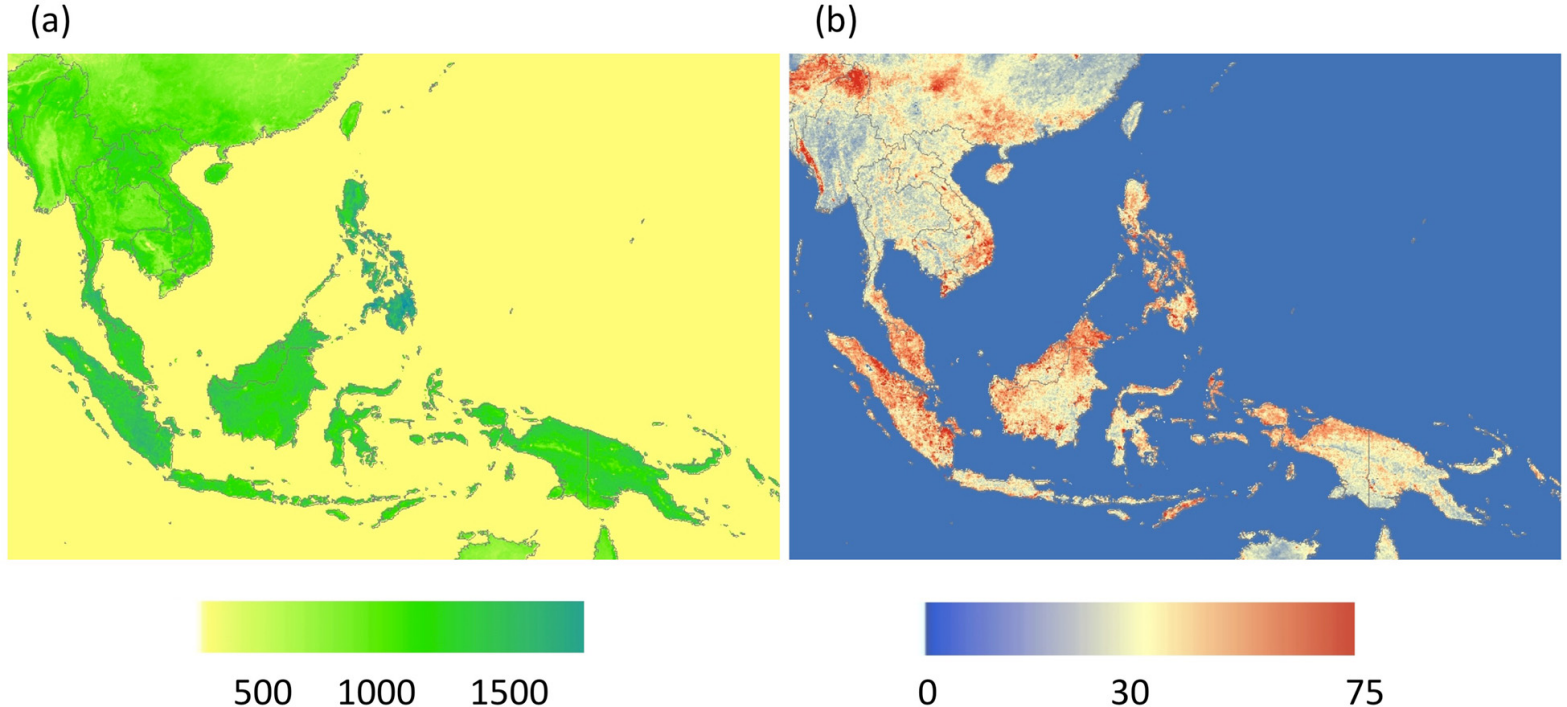
**Regional maps of (a) mean annual NPP and (b) the standard deviation of mean annual NPP from 2000 to 2011 from the CASA model.**All fluxes were reported in units of g C m^-2^ yr^-1^.

**Figure 2:**
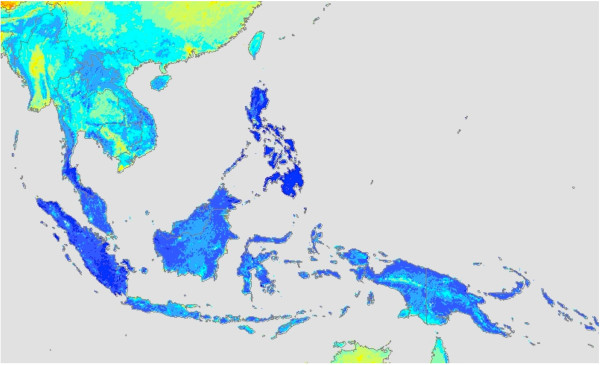
**CASA mean NPP flux over the years 2000 to 2010 classified by natural breaks (Jenks’ optimization).** Legend: Light blue – Low production forest, Medium blue - Moderate production forest, Dark blue – High production forest.

Maps of mean annual NPP and standard deviation of annual NPP (2000 – 2010) showed specific forest areas within these countries with the highest estimated NPP at greater than 1100 g C m^-2^ yr^-1^ (Figure 1a). These areas included the southern Philippines, particularly the eastern and southern sections of the island of Mindanao, the Khao Luang mountain range in southern Thailand, and most forested areas of Sumatra and western Kalimantan. Classification by natural breaks (Jenks’ optimization) into low, moderate, and high production forest categories delineated these areas more clearly (Figure 2). This categorization method minimized each class’s average deviation from the class mean, while maximizing each class’s deviation from the means of the other groups [9].

Forest areas within these countries with the highest estimated variation from year to year in annual NPP fluxes were detected in western coastal Myanmar, southern Vietnam, central Malaysia, eastern Sumatra, and northeastern Borneo (Figure 1b). The standard deviation of the mean NPP in each of these forested areas exceeded 50 g C m^-2^ yr^-1^.

Estimating the annual biomass increment has been a consistent reporting requirement in all Intergovernmental Panel on Climate Change (IPCC) Guidelines, which imply that above ground biomass can be estimated in an acceptable manner with remote sensing techniques and ecosystem models (IPCC, [10]). To calculate the annual change in carbon stock storage in forests (∆C_LUi_), IPCC Guidelines offer the following equation:

$$\Delta C_{\mathrm{LUi}}=\Delta C_{\mathrm{AB}}+\Delta C_{\mathrm{BB}}+\Delta C_{\mathrm{DW}}+\Delta C_{\mathrm{LI}}+\Delta C_{\mathrm{SO}}+\Delta C_{\mathrm{HWP}}$$

where

∆C_AB_ is the annual change in carbon stock storage in above ground biomass;

∆C_BB_ is the annual change in carbon stock storage in below ground biomass;

∆C_DW_ is the annual change in carbon stock storage in dead wood;

∆C_LI_ is the annual change in carbon stock storage in litter;

∆C_SO_ is the annual change in carbon stock storage in soil organic matter;

∆C_HWP_ is the annual change in carbon stock storage in harvested wood products

If it can be assumed that (∆C_AB_ + ∆C_BB_) = ∆NPP, and that the sum of (∆C_DW_ + ∆C_LI_ + ∆C_SO_) changes slowly and is close to zero over short time periods of less than a decade, then this equation can be simplified and rewritten as:

$$\Delta C_{\mathrm{LUi}}=\mathrm{\Delta NPP}+\Delta C_{\mathrm{HWP}}$$

Although this accounting method for annual change in forest carbon stocks cannot substitute entirely for a valid ground-based sample inventory of total standing wood carbon at any given point in time, it does provide an estimate of the change in forest ecosystem carbon storage on a yearly basis. If the C_HWP_ amount is known over a well-defined management area with a high level of confidence for a given year, then the difference between annual NPP and C_HWP_ fluxes becomes an estimate of the change in total carbon stocks for that forest area during that year.

As long as total annual NPP flux exceeds annual C_HWP_ flux by a factor of between two to three, then a forested area should have a positive carbon stock balance, and a net gain in the inventory of total wood carbon for that year. Therefore, 40% of annual NPP [1] summed over the projected period of forest management activities in a given area can conservatively define the upper limit for the amount of harvested wood products that can be removed and still avoid degradation (net loss) of the total wood carbon stock over that same time period.

Case studies from throughout the Southeast Asia region for the maximum C_HWP_ amount that could be sustainably extracted per year (Table 2) were generated using the CASA model NPP predictions shown in Figure 1. In numerous cases studies derived by IGES [11] reports, emission reduction programs are commonly designed to improve forest management within timber concessions. Sustainable management planning typically has the multiple goals of lowering carbon emissions and improving forest health, while still profiting from harvested wood products. Three of the case study projects from Table 2 were explored in more detail below to illustrate the utility of CASA model predictions for establishing sustainable C_HWP_ limits.

**Table 2:** **Case studies from the Southeast Asia region (Source: IGES, [**[11]**]) for the maximum C**_**HWP **_**amount that could be sustainably extracted per year**

						**Limit for sustainable wood extraction**	
**Project name**	**Location**	**Coordinates**	**Area managed**	**CASA NPP**			
		**(lat, lon dd)**	**(ha)**	**(t C ha**^**-1**^**yr**^**-1**^**)**		**C**_**HWP **_**(Mt C yr**^**-1**^**)**	
				Mean	Stdev	Total	+/−
Leuser Ecosystem Project	Aceh Province, Sumatra, Indonesia	3.9369, 97.1807	1, 920. 00	8.95	0.55	6.70	0.41
Berau Forest Carbon Program	East, Kalimantan, Indonesia	1.9030, 117.1028	800, 000	10.30	0.40	3.21	0.12
Kamula Doso Carbon Project	Western Province, Papua New Guinea	-7.2072, 141.9814	791, 200	10.12	0.35	3.12	0.11
April Salumei Forest project	Eat Sepik Province Papau New Guine	-4.6339, 142.7888	521, 000	9.76	0.38	1.98	0.80
Cat Tien National Park Buffer Zone	Lam dong Province, Vietnam	11.7670, 107.5900	251, 445	8.94	0.57	0.88	0.06
Kalimantan forests & Climate	Central Kalimantan, Indonesia	-2.0417, 114.5364	120, 000	8.97	0.46	0.42	0.02
Community Forestry Iniative	Oddar Meanchey Province, Indonesia	14.3668, 103.4292	61, 826	7.15	0.37	0.17	0.01
Merang Peat Swamp Forest	South Sumatra Province, Indonesia	-2.0000, 104.0030	24, 000	10.79	0.88	0.10	0.01

Projects are listed by potential area under management, highest to lowest. Units for NPP are tons carbon per hectare per year (t C ha^-1^ yr^-1^) and for maximum C_HWP_ are million tons carbon per year (Mt C yr^-1^).

In the Berau District, East Kalimantan, Indonesia, The Nature Conservancy has been working since 2006 to promote sustainable harvesting practices through the Responsible Asian Forestry and Trade Program (RAFT). Eight of the district’s 13 timber concessions are working with the Conservancy to improve their forest management by setting aside high conservation value forests, adopting reduced impact logging techniques, and tracking their timber. A stated goal of the project is to avoid emissions of 10 million tons (Mt) of CO_2_ over five years. Conversion from Mt CO_2_ equivalent to Mt C (by the factor of 12/44) puts the goal at about 2.7 Mt C in avoided forest carbon emissions from the project area. The estimated sustainable maximum C_HWP_ amount of 3.2 Mt C yr^-1^ from the CASA model appears to be compatible with this avoided emissions goal.

The Oddar Meanchey Province Community Forestry Initiative for Carbon and Biodiversity Conservation and Poverty Reduction in northern Cambodia estimated that sustainable management and forest protection would result in a net annual stock change of 0.03 Mt C (about 0.11 Mt CO_2_ equivalent yr^-1^) over the first 10 years of the project. This is well below the estimated sustainable maximum C_HWP_ amount of 0.17 Mt C yr^-1^ from the CASA model for this project area.

The Leuser Ecosystem in Aceh Province, Sumatra, Indonesia is considered to be one of the last places in Southeast Asia of sufficient size and quality to maintain viable populations of many rare animal species such as tigers, orangutans, rhinos, elephants, and clouded leopard.

Under Indonesian law, it is illegal to undertake any activities inside the Leuser Ecosystem that are not directly related to either the protection or restoration of the ecosystem. The main driving forces of forest loss in the province include poorly controlled infrastructure development, mining, and conversion to tree crop plantations, namely palm oil.

In management planning reports for the area (IGES, [11]), carbon stocks of forests were estimated at an average of 760 t CO_2_ equivalent ha^-1^ (207 t C ha^-1^). With an estimated 1,920,000 ha of forested land in the Leuser Ecosystem, this totals to roughly 396 Mt C in projected carbon stock, and an average of 17 Mt C yr^-1^ in NPP from the CASA model. Consequently, the sustainable maximum C_HWP_ from the CASA model would be 6.7 Mt C yr^-1^ for the entire Leuser Ecosystem area.

In summary, we have shown in this study that MODIS satellite data has been collected at the broad scale required for useful national estimates of forest carbon production. The daily repeat frequency of MODIS data acquisitions makes this sensor essential to overcome persistent cloud cover for observations of the land surface in tropical Southeast Asia. We have also used Landsat imagery in the same way with the CASA model to monitor forest production and potential degradation at finer scales, such as described in the study by Potter et al. [12].

## Conclusions

•The method of using CASA model’s estimated annual change in forest carbon on a yearly basis can conservatively define the upper limit for the amount of harvested wood products that can be removed and still avoid degradation (net loss) of the total wood carbon stock over that same time period.

•The countries with the highest average forest NPP over the period 2000 to 2010 were the Philippines, Malaysia and Indonesia.

•Highest estimated NPP (at greater than 1100 g C m^-2^ yr^-1^) was detected in the southern Philippines, particularly the eastern and southern sections of the island of Mindanao, the Khao Luang mountain range in southern Thailand, and most forested areas of Sumatra and western Kalimantan.

## Methods

Monthly NPP of vegetation from the CASA model was predicted using the relationship between greenness reflectance properties and the fraction of absorption of photosynthetically active radiation (fPAR), assuming that net conversion efficiencies of PAR to plant carbon can be approximated for different ecosystems or are nearly constant across all ecosystems [13-15]. For this study, we used MODIS collection 5 of the Enhanced Vegetation Index (EVI; Huete, et al. [16]) as model inputs for PAR interception, aggregated for regional assessments of cloud-free imagery to an 8-km spatial resolution.

As documented in Potter [17], monthly production of plant biomass is estimated as a product of time-varying surface solar irradiance, Sr, and EVI (for fPAR) from the MODIS sensor, plus a constant light utilization efficiency term (emax) that is modified by time-varying stress scalar terms for temperature (T) and moisture (W) effects (Equation 1).

(1) $$\mathrm{NPP}=S_{r}\;\mathrm{EVI}\;e_{\max}T\;W$$

The CASA emax term was set uniformly at 0.55 g C MJ^-1^ PAR, an approach that derives from calibration of predicted annual NPP to previous field estimates [3]. This model setting has been successfully validated globally by comparing predicted annual NPP to more than 1900 field measurements of NPP [4], and against numerous Fluxnet eddy covariance tower site measurements of NPP from 2000–2007 [5]. Gridded monthly climate inputs for these CASA runs were from National Center for Environmental Prediction (NCEP) reanalysis products (version NCEP/DOE II; [18]).

## Competing interests

The authors declare that they have no competing interests.

## Authors’ contributions

All authors have made substantial contributions to the acquisition of data, analysis and interpretation of results, drafting the manuscript, and have given final approval of the version to be published.
